# Adaptive local learning in sampling based motion planning for protein folding

**DOI:** 10.1186/s12918-016-0297-9

**Published:** 2016-08-01

**Authors:** Chinwe Ekenna, Shawna Thomas, Nancy M. Amato

**Affiliations:** Department of Computer Science and Engineering, Texas A&M University, College Station, 77843 TX USA

**Keywords:** Protein folding, Motion planning, Machine learning

## Abstract

**Background:**

Simulating protein folding motions is an important problem in computational biology. Motion planning algorithms, such as Probabilistic Roadmap Methods, have been successful in modeling the folding landscape. Probabilistic Roadmap Methods and variants contain several phases (i.e., sampling, connection, and path extraction). Most of the time is spent in the connection phase and selecting which variant to employ is a difficult task. Global machine learning has been applied to the connection phase but is inefficient in situations with varying topology, such as those typical of folding landscapes.

**Results:**

We develop a *local* learning algorithm that exploits the past performance of methods within the neighborhood of the current connection attempts as a basis for learning. It is sensitive not only to different types of landscapes but also to *differing regions* in the landscape itself, removing the need to explicitly partition the landscape. We perform experiments on 23 proteins of varying secondary structure makeup with 52–114 residues. We compare the success rate when using our methods and other methods. We demonstrate a clear need for learning (i.e., only learning methods were able to validate against all available experimental data) and show that local learning is superior to global learning producing, in many cases, significantly higher quality results than the other methods.

**Conclusions:**

We present an algorithm that uses *local* learning to select appropriate connection methods in the context of roadmap construction for protein folding. Our method removes the burden of deciding which method to use, leverages the strengths of the individual input methods, and it is extendable to include other future connection methods.

## Background

Modeling the protein folding process is crucial in understanding not only how proteins fold and function, but also how they misfold triggering many devastating diseases (e.g., Mad Cow and Alzheimer’s [1]).

Knowledge of the stability, folding, kinetics, and detailed mechanics of the folding process may help provide insight into how and why the protein misfolds. Since the process is difficult to experimentally observe, computational methods are critical.

Traditional computational approaches for generating folding trajectories such as molecular dynamics [2], Monte Carlo methods [3], and simulated annealing [4] provide a single, detailed, high-quality folding pathway at a large computational expense. As such, they cannot be practically used to study global properties of the folding landscape or to produce multiple folding pathways. The use of massive computational resources, such as tens of thousands of PCs in the Folding@Home project [5, 6] have helped improve the time overhead involved but still are unable to handle very large proteins. Statistical mechanical models have been applied to compute statistics related to the folding landscape [7, 8]. While computationally more efficient, they do not produce individual pathway trajectories and are limited to studying global averages of the folding landscape.

Robotics-based motion planning techniques, including the Probabilistic Roadmap Method (PRM), have been successfully applied to protein folding [9–11]. They construct a roadmap, or model, of the folding landscape by sampling conformations and connecting neighboring ones together with feasible transitions using a simple local planner. They can generate multiple folding pathways efficiently (e.g., a few hours on a desktop PC) enabling the study of both individual folding trajectories and global landscape properties.

While promising, making good choices for each of the algorithmic steps remains difficult. Machine learning approaches have been used to dynamically decide which approach to take for generating samples and connecting them together. These approaches generally learn *globally* and can perform well in homogeneous spaces or partitioned spaces where each partition is homogeneous [12]. Preliminary work applied connection learning to protein folding simulations [13], but with no way to ensure a good partitioning of the landscape, the results were only comparable to methods with no learning involved.

We present *Local Adaptive Neighbor Connection* (ANC-local) that localizes learning to within the vicinity of the current conformation being connected. When choosing a connection method (i.e., the neighbor selection method and local planner combination), we first dynamically determine a neighborhood around the conformation under consideration. Then, the performance history within this neighborhood is used to bias learning. Our method adapts *both* over time and to local regions without any prior knowledge about the methods involved. This approach has been successfully used in robotics [14], and here we adapt it to protein folding.

We compare ANC-local’s performance to three distance-based connection methods and to global learning over 23 proteins of varying secondary structure makeup with 52–114 residues. We examine both the time to build roadmaps and the resulting trajectory quality. We further look at the local planner success rate to understand performance changes between methods. Our results confirm that learning is necessary, as no individual method is the best choice for all proteins. We also show that ANC-local generates better quality trajectories in comparable time than the best connection method for each individual input and outperforms global learning.

We next describe some preliminaries and related work in further detail, including experimental protein dynamics, the protein model used, PRMs for protein folding, and several key components such as candidate neighbor selection methods and distance metrics. We also discuss existing machine learning techniques for PRMs and for protein motion and analysis.

### Experimental protein dynamics

There have been several advances in experimental techniques to study protein dynamics and motion including circular dichroism, fluorescence experiments, hydrogen exchange and pulse labeling, NMR spectroscopy, and time-resolved X-ray crystallography. We briefly discuss each in turn.

Circular dichroism (CD) is a spectroscopic technique used to investigate the structure and conformational changes of proteins [15]. By informing on binding and folding properties, CD provides information about the protein’s biological functions. The CD signal occurs when chromophores in an asymmetrical environment interact with polarized light. In the case of proteins, the main chromophores are the peptide bonds as they absorb polarized light in the far-UV wavelength region (i.e., below 240 nm).

Fluorescence spectroscopy analyzes the emission of fluorophores in the protein as the protein undergoes conformational change [16], such as during folding or upon binding. These fluorophores act as indicators of the state of the local environment, e.g., how structured the portion of the protein is near the fluorophore. As almost all proteins have natural fluorophores (i.e., tyrosine and tryptophan residues), fluorescence spectroscopy has broad applicability.

Hydrogen exchange mass spectrometry and pulse labeling can investigate protein folding by identifying which parts of the structure are most exposed or most protected [17]. From this data, one can infer which portions of the protein fold first and which are last to form, up to the millisecond timescale.

NMR spectroscopy, another experimental tool often used to study protein dynamics, is a technique used to determine a compound’s unique structure. It identifies the carbon-hydrogen framework of an organic compound and has been used to study side-chain motion and backbone motion [18]. See [19] for a recent review of current techniques.

X-ray crystallography obtains a three dimensional molecular structure from a crystal [20]. A purified sample at high concentration is crystallized and the resulting crystals are exposed to an x-ray beam. This produces a pattern of diffraction spots. The intensities of these spots can be used to determine the structure factors from which an electron density map can be calculated.

While experimental methods can probe some fine-grained details of protein motion, they are time intensive and limit the time scales they can access. In addition, experimental methods may not be able to be applied to all proteins, e.g., some proteins naturally precipitate out and cannot be analyzed. Simulations, instead, affords the opportunity to study such proteins and others much faster (hours vs. days) with computational resources which will potentially save both time and money.

### Protein model

Proteins are sequences of amino acids, or residues. We model the protein as a linkage where only the *ϕ* and *ψ* torsional angles are flexible, a standard modeling assumption [21]. A potential energy function models the many interactions that affect the protein’s behavior [2]. This function helps quantify how energetically feasible a given conformation is.

In this work, we employ a coarse-grained potential function [9] which help define some characteristics of our modeling and they state that- If the atoms are too close to each other (less than 2.4Å in sampling and 1.0Å in connecting), the conformation is unfeasible; otherwise, the energy is calculated by:

1 $$ U_{tot} = \sum\limits_{constraints}K_{d}\{[(d_{i} - d_{0})^{2} + {d_{c}^{2}}]^{1/2} - d_{c}\} + E_{hp}  $$

where *K*_*d*_ is 100 kJ/mol, *d*_*i*_ is the length on the *i*th constraint, *E*_*hp*_ is the hydrophobic interaction, and *d*_0_=*d*_*c*_=2Å as in [2]. The coarse grain model has been shown to produce qualitatively similar results as all-atoms models faster [22].

### PRM for protein folding

The Probabilistic Roadmap Method (PRM) [23] is a robotics motion planning algorithm that first randomly samples robot (or protein) conformations, retains valid ones, and then connects neighboring samples together with feasible motions (or transitions). To apply PRMs to proteins, the robot is replaced with a protein model and collision detection computations are replaced with potential energy calculations [9–11, 24].

#### Sampling

Protein conformations, or samples, are randomly generated with bias around the native state, the functional and most energetically stable state. Samples are iteratively perturbed, starting from the native state, and retained if energetically feasible by the following probability:

2 $$ P(q) =\left\{ \begin{array}{ll} 1 & \text{if}~E(q) < E_{min} \\ \frac{E_{max} - E(q)}{E_{max} - E_{min}} & \text{if}~ E_{min} < E(q) \leq E_{max} \\ 0 & \text{if}~E(q) > E_{max} \end{array} \right.  $$

where *E*_*min*_ is the energy of the open chain and *E*_*max*_ is 2 *E*_*min*_. We use rigidity analysis to focus perturbations on flexible portions as detailed in [25].

#### Connection

Once a set of samples is created, they must be connected together with feasible transitions to form a roadmap, or model of the folding landscape. Connecting all possible pairs of samples is computationally unfeasible, and it has been shown that only connecting the *k*-closest neighbors results in a roadmap of comparable quality [26].

Given a pair of samples, we compute a transition between them by a straight-line interpolation of all the *ϕ* and *ψ* torsional angles. Straight-line local planning involves the fewest number of intermediates to check for validity and has been shown to be a sufficient measure of transition probability; i.e., it can accurately predict secondary structure formation order [9, 22].

We assign an edge weight to reflect the energetic feasibility of the transition as $\sum _{i=0}^{n-1}-log(P_{i})$ where *P*_*i*_ is the probability to transit from intermediate conformation *c*_*i*_ to *c*_*i*+1_ based on their energy difference *ΔE*_*i*_=*E*(*c*_*i*+1_)−*E*(*c*_*i*_):

3 $$ P_{i} =\left\{ \begin{array}{ll} e^{\frac{-\Delta E_{i}}{kT}} & \text{if}~\Delta E_{i} > 0 \\ 1 & \text{if}~\Delta E_{i} \leq 0 \end{array} \right.  $$

where *k* is the Boltzmann constant and *T* is the temperature. This allows the most energetically feasible paths to be extracted by standard shortest path algorithms.

#### Validation by secondary structure formation order

Proteins are composed of secondary structure elements (i.e., *α*-helices and *β*-strands). Experimental methods, such as hydrogen exchange mass spectrometry and pulse labeling, can investigate protein folding by identifying which parts of the structure are most exposed or most protected [27]. From this data, one can infer the secondary structure formation order.

In [9, 21, 22], we compared the secondary structure formation order of folding pathways extracted from our maps to experimental results [28] by clustering paths together if they have the same formation ordering. We return a stable roadmap when the distribution of secondary structure formation orderings along the folding pathways in the graph stabilizes, i.e., the percentage of pathways following a given ordering does not vary between successive graphs by more than 30 %. As our roadmaps contain multiple pathways, we estimate the probability of a particular secondary structure formation order occurring by the percentage of roadmap pathways that contain that particular formation order. The roadmap corroborates experimental data when the dominant formation order (i.e., the one with the greatest percentage) is in agreement.

### Candidate neighbor selection methods

Recall that only neighboring (or nearby) samples are attempted for connection because it is unfeasible to attempt all possible connections. Typically, conformations that are more similar are more energetically feasible to connect.

There have been a number of methods proposed for locating candidate neighbors for connection. The most common is the *k*-closest method which returns the *k* closest neighbors to a sample using a distance metric. This can be implemented in a brute force manner taking *O*(*k* log*n*)-time per node, totaling *O*(*nk* log*n*)-time for connection. A similar approach is the *r*-closest method which returns all neighbors within a radius *r* of the node as determined by some distance metric.

Other methods use data structures to more efficiently compute nearest neighbors. *Metric Trees* [29] organize the nodes in a spatial hierarchical manner by iteratively dividing the set into two equal subsets resulting in a tree with *O*(log*n*) depth. However, as the dataset dimensionality increases, their performance decreases [30]. *KD-trees* [31] extend the intuitive binary tree into a D-dimensional data structure which provides a good model for problems with high dimensionality. However, a separate data structure needs to be stored and updated.

Approximate neighbor finding methods address the running time issue by instead returning a set of approximate *k*-closest neighbors. These include spill trees [30], MPNN [32], and Distance-based Projection onto Euclidean Space [33]. These methods usually provide a bound on the approximation error.

In this paper, we work with proteins with a higher dimensionality (104 to 228 degrees of freedom) than approximate methods can handle. Note, however, that there is nothing inherent in our approach that precludes the use of approximate methods.

### Distance metrics

The distance metric plays an important role in determining the best connections to attempt. It is a function *δ* that computes some “distance” between two conformations *a*=〈*a*_1_,*a*_2_,…,*a*_*d*_〉 and *b*=〈*b*_1_,*b*_2_,…,*b*_*d*_〉, i.e., $\delta (a, b) \rightarrow \mathbb {R}$, where *d* is the dimension of a conformations. Here, *a*_1_… and *b*_1_… are the *ϕ* and *ψ* torsional angles for each protein conformation. A good distance metric generally predicts how likely it is that a pair of nodes can be successfully connected. Their success is dependent on the nature of the problem studied. We use the following set of distance metrics commonly used for motion planning:

#### Euclidean distance metric

The Euclidean distance metric captures the amount of physical movement (around the torsional angles) that conformation *a* would undertake to move to a conformation *b*. This distance is computed by measuring the difference in the *ϕ* and *ψ* angle pairs of the two conformations:

4 $$ {{} {\begin{aligned} \delta_{\text{Eucl}}(a,b) = \sqrt{\frac{\left({{\phi_{1}^{a}} - {\phi_{1}^{b}}}\right)^{2} + \left({{\psi_{1}^{a}} - {\psi_{1}^{b}}}\right)^{2} +... + \left({{\phi_{n}^{a}} - {\phi_{n}^{b}}}\right)^{2} + \left({{\psi_{n}^{a}} - {\psi_{n}^{b}}}\right)^{2}}{2n}}. \end{aligned}}}  $$

#### Cluster rigidity distance metric

Rigidity analysis [34] computes which parts of a structure are rigid and flexible based on the constraints present. It may be used to define a rigidity map *r*, which marks residue pairs *i,j* if they are in the same rigid cluster.

Rigidity maps provide a convenient way to define a rigidity distance metric, between two conformations *a* and *b* where *n* is the number of residues:

5 $$ \delta_{\text{Rig}}(a,b) = \sum\limits_{0 \leq i < j \leq 2n} (r_{a}(i,j) \neq r_{b}(i,j)).  $$

More details may be found in [25].

#### Root mean square distance metric

The protein model has 6 atoms for each amino acid. Thus, a protein with *n* amino acids will have 6*n* atoms. Denoting the coordinates of these atoms as *x*_1_ to *x*_6*n*_, the root mean square distance (RMSD) between conformations *a* and *b* is:

6 $$ {{} {\begin{aligned} \delta_{\text{RMSD}}(a,b)=\sqrt{\frac{\left({{x_{1}^{a}} - {x_{1}^{b}}}\right)^{2} + \left({{x_{2}^{a}} - {x_{2}^{b}}}\right)^{2} +... + \left({x_{6n}^{a} - x_{6n}^{b}}\right)^{2}}{6n}}. \end{aligned}}}  $$

Least RMSD (lRMSD) is the minimum RMSD over all rigid body superpositions of *a* and *b*.

### Machine learning for protein analysis and motion

Machine learning algorithms have been employed to predict protein folds, estimate folding rates, and study folding motions. We highlight a few relevant techniques here.

#### Protein fold recognition

Protein fold recognition involves identifying the correct structural fold from among a set of known template protein structures for a given protein sequence. Fold recognition is essential for template-based protein structure modeling. The fold recognition problem is defined as a binary classification problem of predicting whether or not the unknown fold of the input protein is similar to an already known template from a protein structure library.

RF-Fold uses random forests, a highly scalable classification method, to recognize protein folds [35]. A random forest is composed of many decision trees that are each trained on datasets of target-template protein pairs. RF-Fold recognition rate is comparable to the best performance in fold recognition at the family, superfamily, and fold levels.

DN-Fold is another fold recognition technique, but it uses a deep learning neural network as a basis for learning [36]. A deep learning network has many more layers than a typical neural network. In addition, they may be trained through unsupervised learning. Deep learning was applied to fold prediction by restating the problem as predicting if a given target-template pair belonged to the same fold. They showed that DN-Fold achieved comparable performance over a wide variety of methods at all three fold levels.

#### Folding rate prediction

In addition to predicting the fold of a protein, it is useful to estimate its folding rate. This is important when studying properties such as stability and classifying kinetics. Characteristics of the protein structure, such as contact order and total contact distance, affect the folding rate. However, the precise relationship between these characteristics and the rate are unknown. A back-propagation neural network was used to quantify this relationship [37]. Their results showed that correlations exist between these properties and the folding rate with relative errors for predicted results lower than competing methods.

#### Simulating protein motion trajectory

Machine learning has also been applied to studying protein folding trajectories. In [38] they use unsupervised learning to cluster similar states and basins present in the folding landscape. They then use this clustering to construct an exploration bias to speed up molecular dynamics simulations. Specifically, the exploration bias guides the next basin to jump to in the simulation while ensuring that the entire conformation space is explored. They provide simulation results for an alanine trajectory.

### Machine learning for PRMs

Many techniques use machine learning to improve PRM performance. In this section we briefly highlight some of these methods.

#### Learning sampling methods

In Feature Sensitive Motion Planning [39], the planning space is recursively subdivided and machine learning is used to characterize the resulting partitions and select an appropriate PRM variant to use in each. A key strength of this approach is its ability to map workspace/C-Space topologies for planners to work in. However, it does not adapt planner choices over time.

HybridPRM [40] uses reinforcement learning to adaptively select the appropriate sampling method over time. It does so by maintaining a selection probability for each method and updates these probabilities based on the method’s past performance. While learning adapts over time, it is global. It does not perform well when the planning space is heterogeneous, as is the case for most protein folding landscapes.

RESAMPL [12] is similar in spirit to Feature Sensitive Motion Planning, but it dynamically generates local regions to plan in. Instead of using supervised learning, it uses local region information (e.g., entropy of neighboring samples) to make decisions about how and where to sample, and which samples to connect together.

While the classification of a region may change over time as it is explored, it’s placement does not. Thus it cannot adequately adapt if the initial region placement or resolution is not sufficient.

#### Learning connection methods

Prior work [41] adaptively selects the appropriate connection method to use over time. As the roadmap is built, it records the performance of several connection methods and with this history, decides which to employ by maintaining a selection probability for each. The main weakness of Adaptive Neighbor Connection (ANC-global) is that it bases its decisions on the performance of connection methods over the *entire* planning space. This is problematic in protein landscapes that are naturally heterogeneous. Therefore, to obtain better results, it became necessary to first partition the space into smaller (and hopefully homogeneous) regions. This puts greater burden on the user, particularly as the dimensionality of the problem increases. While ANC-global was applied to proteins, its performance was limited and so a *local* learning approach is needed.

#### Learning from trajectories

Some methods have been proposed to learn from previous experience. For example, the Lightning framework [42] executes two components in parallel: a traditional planning from scratch approach and an approach that extracts and repairs paths from a path history library. It uses the result of the fastest component as the final solution and then adds it to the path history library for future use. Note that as the size of the library grows, it becomes impractical to add additional paths to it.

Apprenticeship Learning [43] also uses existing trajectories to plan motion, but instead aims to learn good trade-offs between different cost functions that describe properties of the trajectories. It learns these trade-offs via inverse reinforcement learning The premise is to learn from a small set of demonstration trajectories instead of a large path library.

## Methods

Our learning framework is a machine learning reinforcement learning method that stems from multi-armed bandit problem algorithms [44, 45]. In the multi-armed bandit problem, the goal is to find the arm (action) with the highest expected payoff during a gambling game of cards as soon as possible and then keep gambling using that best arm. Each selected arm is associated with a reward, and the gambler’s objective is to maximize his cumulative expected earnings during the game duration. To do this, the gambler needs to acquire information about arms (exploration) while simultaneously optimizing immediate rewards (exploitation).

We apply this to selecting which connection method to use for a given protein sample/conformation by redefining the reward and cost functions of choosing a connection method. As in the multi-armed bandit problem, we aim to maximize connection success while also exploring other methods that may perform well later on in the connection process.

### The local learning approach

In *Local Adaptive Neighbor Connection* (ANC-local), learning is localized to within the vicinity of the current conformation being connected. When choosing a connection method, the current conformation’s neighborhood is dynamically determined. This neighborhood is defined as the set of nearest neighbors given by some distance metric.

We use the performance history of only those connection attempts within this neighborhood to bias learning. Thus, our method adapts both spatially and temporarily, and no prior knowledge about the connection method involved is needed. This approach has been introduced for robotic motion planning [14], and here we adapt it to simulate the folding process.

For proteins, we measure performance as a function of the edge weights in the roadmap and the time needed to construct a stable roadmap. We want to balance both compute time and trajectory quality where quality may be inferred from the edge weights (i.e., their energetic feasibility). Performance is measured only from the dynamically determined neighborhood so learning is continuous and localized.

### Example

**Fig. 1:**
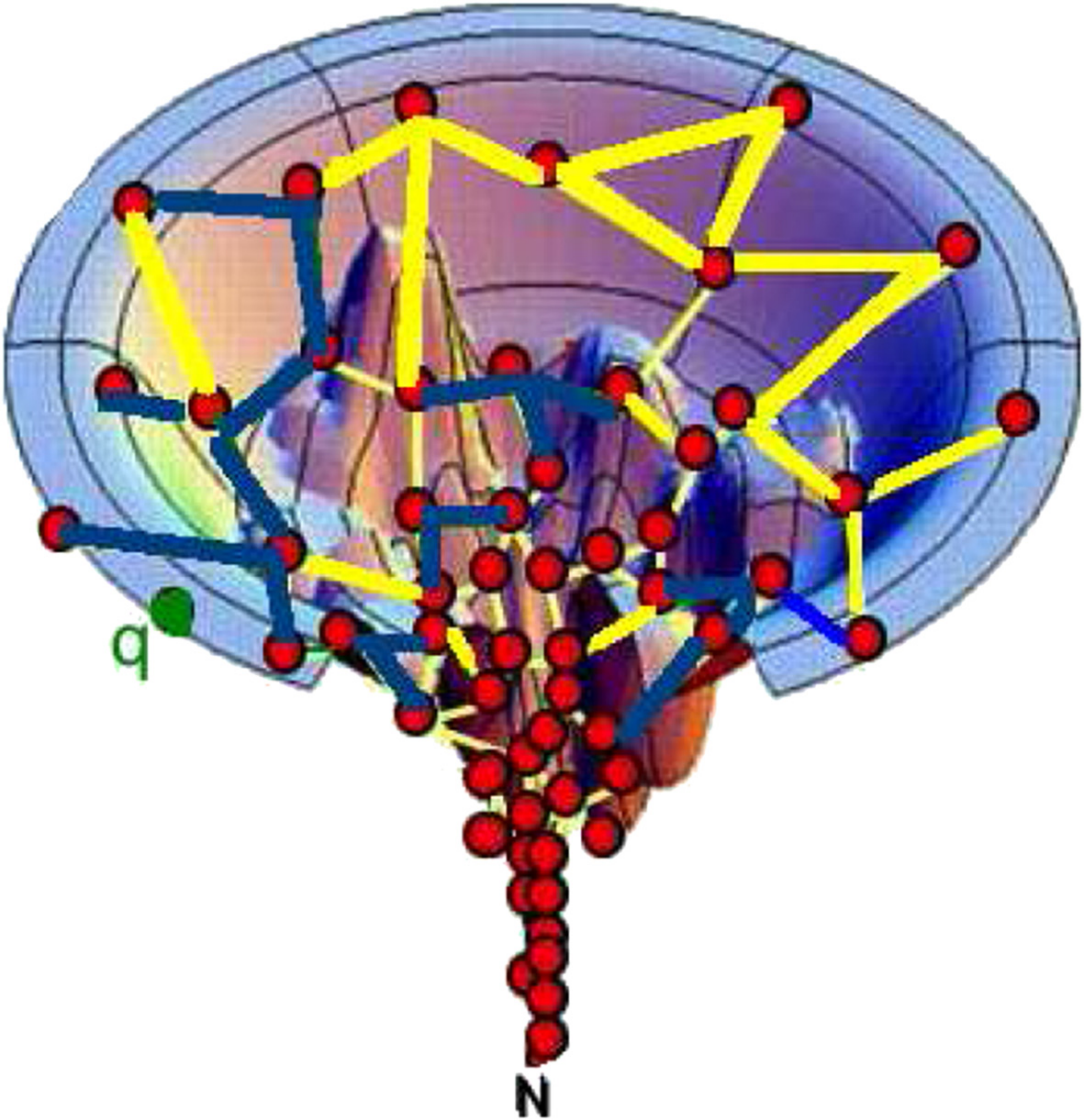
Example energy landscape and roadmap. Two connection methods are used to build a roadmap on the protein’s energy landscape: *C* *M*_*A*_ (*yellow/light*) and *C* *M*_*B*_ (*blue/dark*). When connecting a new conformation *q* (*in blue*), it is important to learn from local information not global, as *C* *M*_*B*_ is more locally successful even though a majority of the edges are from *C* *M*_*A*_

Figure 1 shows an example energy landscape and roadmap. The roadmap is constructed with two candidate connection methods: *CM*_*A*_ (yellow/light) and *CM*_*B*_ (blue/dark). Edges added by *CM*_*A*_ are yellow/light, and those added by *CM*_*B*_ are blue. Overall, the most successful connection method is *CM*_*A*_ (with more yellow/light edges). However, in the left region of the landscape, *CM*_*B*_ is much more successful. When connecting node *q* (in green) to the roadmap, it is important to take locality into account. A global learning method, such as ANC-global, would select *CM*_*A*_ to connect *q*, but this would be a poor choice. A local learning method, such as ANC-local, would instead choose *CM*_*B*_ to connect *q* because *CM*_*B*_ is more successful there.

### Method details

Algorithm 1 describes the ANC-local algorithm as introduced in [14]. We initialize all the methods *M* to the uniform probability and determine the local learning region as defined by the set of nearest neighbors using *NF*_*local*_ in *D*, where *D* is a tuple containing the connection method, reward, and cost. For each determined neighbor, we update the probability using the UpdateProbability function in Algorithm 2 and make a connection based on the chosen connection method *cm*.





The UpdateProbabilty function (Algorithm 2) is used to continually calculate and update the probabilities of the connection methods. This is where performance is monitored and reinforcement learning takes place.

Potential energy computations take up a large portion of the total computation time and thus are a good measure of cost. Here, we calculate the cost as the number of potential energy calls incurred by the connection method.

ANC-local maintains a weight for each connection method similar to Hybrid PRM [40] but reconstructed to handle potential energy calculations. These weights keep track of the past performance of each connection method. ANC-local initializes each weight *w*_*i*_ to 1. Based on the weights, ANC-local computes in a step-wise manner a probability $p^{*}_{i}$ for *cm*_*i*_ without considering the cost:

7 $$ p^{*}_{i} = (1-\gamma)\frac{w_{i}(t)}{\sum\limits_{j=1}^{m} w_{j}(t)} + \gamma \frac{1}m, i=1,2,..., m,  $$

where *w*_*i*_(*t*) is the weight of *cm*_*i*_ in step *t*, *t* is the current connection attempts made, and *γ* is a fixed constant. The probability $p^{*}_{i}$ is a weighted sum of the relative weight of *cm*_*i*_ and the uniform distribution. This ensures that each connection method has some chance of being selected.

Let *x*_*i*_ be the reward for the *cm*_*i*_ that was selected:

8 $$ x_{i} =\alpha + (1-\alpha)(1-\frac{{y_{i}(t) - miny_{i}(t)}}{maxy_{i}(t) -miny_{i}(t)})  $$

where *y*_*i*_(*t*) = current edge weight, *miny*_*i*_(*t*) = minimum edge weight recorded during the current step, *maxy*_*i*_(*t*) = maximum edge weight recorded during the current step, and *α* = a constant value used to normalize the reward. All other rewards for that time step are 0. The reward is thus a function of the edge quality (weight) and the local planner’s success.

To update the weights, we first take into account an adjusted reward that is not dependent on the cost accrued:

9 $$ x^{*}_{i} = x_{i}/p^{*}_{i}, i=1,2,...m.  $$

Then we update the weights for all the connection methods:

10 $$ w_{i}(t+1) = w_{i}(t)\exp{\frac{{\gamma x^{*}_{i}}}m}, i=1,2,...m.  $$

The new weight is the current weight multiplied by a factor that depends on the reward received. The exponential factors enable the weights to adapt quickly.

We now include the cost in the selection probability:

11 $$  p_{i} = \frac{\frac{p^{*}_{i}}{c_{i}}}{{\sum\limits_{j=1}^{m}}\frac{p^{*}_{i}}{c_{j}}}, i=1,2,...K.  $$

where *c*_*i*_ is the average cost of attempting to connect *i*.

## Results and discussion

In this section, we investigate the performance of ANC-local (local learning), ANC-global (global learning), and individual connection methods to model the folding landscape of 23 proteins. Individual connection methods are *k*-closest neighbor selection using either Cluster, Euclidean, or lRMSD distance metric. ANC-global and ANC-local use these methods as their learning set.

We first establish each method’s ability (individual connection methods, global learning, and local learning) to validate against experimental data when available. We then look into the local planner success rate in the context of each strategy. We examine the quality of the resulting folding pathways and the time required by each individual method and look at the cumulative performance of these metrics. We show how ANC-local’s learning decisions corroborate with the individual connection method performance outside of the learning framework. In addition, we compare ANC-local’s learning performance against ANC-global’s learning approach.

### Experimental setup

We study 23 proteins (see Table 1) with 52–114 residues. This set contains *α*, *β*, and mixed proteins that were also studied by [46] and many have experimentally determined secondary structure formation orders [47]. The protein structures were obtained from the Protein Data Bank [48].

**Table 1:** Proteins studied

Protein name	PDB ID	Length	Secondary structure	
Rubredoxin	1RDV	52	2*α*+2*β*	
Ferredoxin	1FCA	55	2*α*+2*β*	
Protein G	1PGA	56	1*α*+4*β*	
Protein G Variant	NUG1	57	1*α*+3*β*	
Protein G Variant	NUG2	57	1*α*+3*β*	
Alpha-Spectrin SH3 Domain	1SHG	57	1*α*+5*β*	
Human FYN	1NYF	58	5*α*+1*β*	
Immunoglobulin G	2SPZ	58	3*α*	
Binding Protein A				
Cardiotoxin III	2CRS	60	5*β*	
Tick Antocoagulant peptide	1TCP	60	2*α*+2*β*	
ADR1	2ADR	60	2*α*+2*β*	
Repressor Protein C1	1R69	63	5*α*	
Chymotrypsin Inhibitor 2 variant	1COA	64	1*α*+4*β*	
Chymotrypsin Inhibitor 2 variant	2CI2	65	1*α*+4*β*	
Probable enterotoxin	2KRS	70	7*β*	
Regulatory Protein CRO	2CRO	71	5*α*	
Protein L	2PTL	78	1*α*+4*β*	
Procarboxy peptidase B	1PBA	81	4*α*+3*β*	
Procarboxy peptidase A2	106X	81	2*α*+3*β*	
ACYL-CO Enzyme	2ABD	86	4*α*	
Barnase	1YVS	106	3*α*+4*β*	
Binase	1BUJ	109	5*α*+3*β*	
DNA B Helicase	1JWE	114	8*α*	

For all experiments, we generate conformations using iterative sampling based on rigidity analysis [25]. For all connection methods, we use a straight line local planner and attempt to connect to the 20 nearest neighbors. For ANC-local, we set *NF*_*local*_ to be the 40 nearest neighbors based on Euclidean distance. This resulted in the best performance in preliminary experiments. We stop construction once we have a stable roadmap.

Metrics are computed as follows:

- *Secondary structure formation order:* We compare, when available, the secondary structure formation order predicted by each method to experimental data. We examine shortest paths from all unfolded states to the native state. (Recall that roadmap edge weights reflect the transition’s energetic feasibility, so extracting the smallest weighted path corresponds to extracting the most energetically feasible path). We then compare the dominant ordering (i.e., the ordering that occurs most frequently among all folding pathways present) to the ordering given by experimental data.
- *Pathway quality:* We define folding pathway quality as the weight of each edge (i.e., its energetic feasibility) multiplied by the dominance of that edge (i.e., the number of folding pathways that traverse it). This metric is important because it identifies how many edges with low energies are present and how frequently they are used.
  Having low quality values in our results indicate a better performing connection methods.

### Validation by secondary structure formation order

Table 2 summarizes the comparison of each method’s dominant secondary structure formation order. (Entries are ordered as appears in Table 1 by protein length.) Only the learning methods (ANC-global and ANC-local) produced the same dominant formation order as experiment for all proteins with available data. Individual methods were unable to reproduce the ordering from experimental data for 2ABD. Thus, in some cases learning is required for correctness.

**Table 2:** Validation of secondary structure formation order to experimental data when available. Proteins are ordered by protein length as in Table 1

PDB	Experimental					
identifier	data	ANC-	ANC-	Cluster	Euclidean	lRMSD
		local	global			
1RDV	Unavailable	Same ordering				
1FCA	Unavailable	Same ordering				
1PGA	[49]	Y	Y	Y	Y	Y
NUG1	[50]	Y	Y	Y	Y	Y
NUG2	[50]	Y	Y	Y	Y	Y
1SHG	[47, 51]	Y	Y	Y	Y	Y
1NYF	[52, 53]	Y	Y	Y	Y	Y
2SPZ	Unavailable		Different orderings			
2CRS	[28]	Y	Y	Y	Y	Y
1TCP	Unavailable	Same ordering				
2ADR	Unavailable	Same ordering				
1R69	Unavailable	Same ordering				
1COA	Unavailable	Same ordering				
2CI2	[54]	Y	Y	Y	Y	Y
2KRS	[47]	Y	Y	Y	Y	Y
2CRO	Unavailable	Same ordering				
2PTL	[55]	Y	Y	Y	Y	Y
1PBA	Unavailable	Same ordering				
106X	[56]	Y	Y	Y	Y	Y
2ABD	[57]	Y	Y	**N**	**N**	**N**
1YVS	[58]	Y	Y	Y	Y	Y
1BUJ	Unavailable		Different orderings			
1JWE	Unavailable	Same ordering				
# Agree with Exp. / # Available		12/12	12/12	11/12	11/12	11/12

When experimental data was not available, all methods produced the same ordering for 9 proteins and different orderings for 2 proteins (2SPZ and 1BUJ). Upon examination of the 2 proteins that methods disagree on, we find that ANC-local, ANC-global, and Cluster are always in agreement and Euclidean and lRMSD are always in agreement. Additionally, disagreements only occur at the end of the pathway; all methods agree on the order of the first elements to form. Specifically, all methods find that the central *α*-helix forms first in 2SPZ and disagree on the relative ordering of the two terminal *α*-helices. Similarly, all methods find that *β*-strands 6, 5, 4, 3, 2 form first (and in that order) and disagree on the relative ordering of the three *α*-helices and the remaining *β*-strand for 1BUJ.

### Local planner success rate

Recall that a connection method comprises both the distance metric used to identify neighbors to connect and a local planner (e.g., a straight-line in *ϕ*−*ψ* space) that computes a set of intermediate conformations, evaluates their energetic viability, and adds an edge between the two neighbors if such trajectory is feasible. The local planner success rate is a good indicator of the performance of the whole connection process. We measure the local planner success rate as the number of connections made out of the number of connections attempted.

**Fig. 2:**
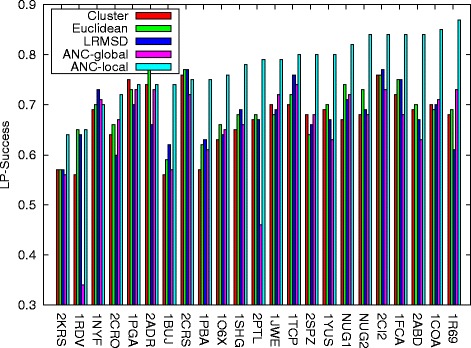
Local planner success rate for each method over all proteins studied. The local planner success rate of ANC-local is greater than all the other methods for 18 of the 23 proteins studied and comparable for 1 of the proteins. Note that entries are ordered by the local planner success rate in the context of ANC-local

Figure 2 displays the local planner success rate for all connection methods across all proteins studied. Observe that the local planner success rate is highest for ANC-local for 18 of the 23 proteins and comparable for 1 of the proteins (1RDV). For proteins in which it is not the highest (1NYF, 1PGA, 2ADR, 2CRS), it is within 0.05 of the highest. Note that ANC-global does not perform as well as ANC-local and in many cases (for 15 proteins it is greater than 0.1 lower) is significantly lower. This indicates that not only is learning important, but *local* learning is crucial to properly adapting to different protein folding landscapes. ANC-local consistently makes wise choices for connection that yield successful local planner attempts, which are quite costly.

### Quality, time, and the tradeoff between them

#### Quality

**Fig. 3:**
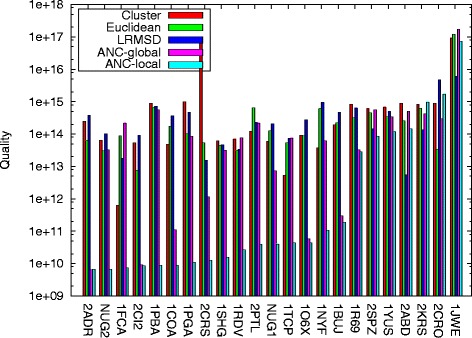
Roadmap quality for each method over all proteins studied. No single individual connection method performs best across all proteins. ANC-local produces the best quality roadmaps for 18 of the 12 proteins studied. Note that entries are ordered by ANC-local performance

Figure 3 shows the resulting folding pathway quality of each connection method, ANC-global, and ANC-local. Entries are ordered by ANC-local performance (and not by protein length). Recall that the aim is to generate pathways with low weight/energy. Only looking at individual connection method performance, we first see that no single connection method performs the best across all proteins: Cluster is the best choice for 7 proteins, Euclidean for 11 proteins, and lRMSD for 5 proteins. In addition, there is no correlation between individual connection method performance and secondary structure makeup or size. Thus, there is a clear need for learning.

It is not surprising then that learning methods outperform the best individual connection methods much of the time: ANC-global (pink bars) produces lower weighted pathways than Cluster, Euclidean, and lRMSD for 11 of the 23 proteins, and ANC-local (blue bars) for 19 of the 23 proteins. Notice, however, that the type of learning is important. ANC-local with its local learning is much more successful than ANC-global with its global approach. ANC-global outperforms ANC-local for only 1 protein in the set (2ADR) and even then the performance is only marginally better while ANC-local outperforms ANC-global by a large margin for many of the proteins. In fact, ANC-local is the best approach for 18 out of the 23 proteins studied. Note that the best performing method in the other 5 proteins is not the same (many of them are at the far right of Fig. 3): lRMSD produces lower weight pathways for 3 proteins (2KRS, 2ABD, and 1JWE), Euclidean for 1 (2CRO), and ANC-global for 1 (2ADR).

Additionally, in 17 of the 18 proteins where ANC-local produces the best quality, it produces significantly better quality than the other methods for 12 of the 18. We see an improvement of ANC-local over ANC-global in terms of quality for 20 of the 23 proteins studied. Of the 3 remaining proteins (2ADR, 2CRO, 2KRS) where ANC-global performs better, ANC-local performance is comparable.

#### Time

**Fig. 4:**
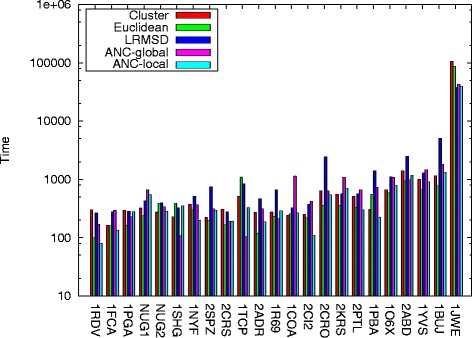
Time for each method over all proteins studied. ANC-local performs as well as or better than the best performing method for 12 out of 23 proteins studied. Note that entries are ordered by protein length

Figure 4 provides the time needed to build stable roadmaps for each method, ordered by protein length. ANC-local is the fastest for 6 of the proteins and the second fastest for 6, with 3 of those incurring less than 10 % overhead. Thus, ANC-local performs as well as or better than the best performing method for 12 out of 23 proteins (52 % of the time), while ANC-global performs best for only 3. Just as with quality, the best performing individual connection method varies between proteins: Euclidean is fastest for 11 proteins, Cluster for 2, and lRMSD for 1. Euclidean is most often the fastest method but is the best method in terms of quality for only 1 protein.

**Fig. 5:**
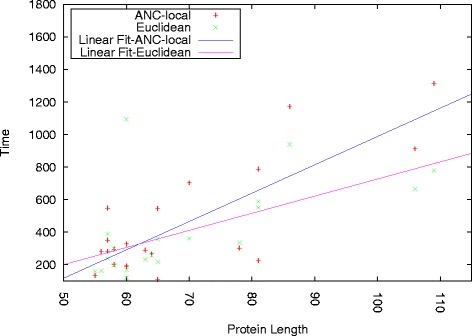
Time as a function of protein length. ANC-local and its fastest competitor, Euclidean, display a roughly linear relationship between time and protein length

To further understand the scalability of these approaches, we plot the time to build a stable roadmap as a function of protein length for both ANC-local and its fastest competitor, Euclidean. Each point in Fig. 5 corresponds to the time taken for a protein of that length. Figure 5 also plots a linear regression for each data set. There is a roughly linear relationship between length and running time (correlation coefficients of 0.55 for ANC-local and 0.53 for Euclidean; higher polynomial regressions fit poorly).

Note that while we see some overhead for learning (i.e., a steeper regression line), other methods may not produce pathways of high quality. For example, ACYL-CO Enzyme (2ABD) is a protein where only ANC-local produced the correct secondary structure formation order as seen in experiment (see Table 2). It is also the furthest outlier above the regression line (length 86). While more time is consumed constructing a stable roadmap for this protein, it is time well-spent as it produces the correct secondary structure formation order while others do not.

#### Quality vs. time

**Fig. 6:**
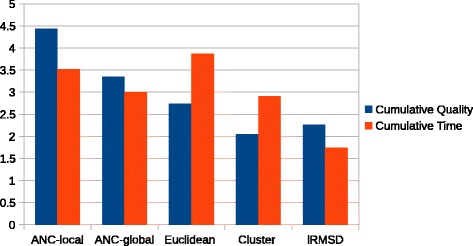
Cumulative performance of each method over all proteins studied. Methods are ranked from 1 (worst) to 5 (best). Entries are ordered by cumulative quality ranking. ANC-local performs better than the other methods across the entire protein set in terms of quality and second best in terms of time

Finally, we look at each method’s cumulative performance to examine how these two metrics interplay. Figure 6 shows the ordered ranking of each connection method, ANC-global, and ANC-local across all 23 proteins. For each protein, we assign a rank from 1 to 5 (with 5 being the best) to each method for quality and time. The cumulative performance for each method is the average of these rankings.

ANC-local performs better than the other connection methods across the entire protein set in terms of quality and second best in terms of time. lRMSD, as expected, is the slowest. While ANC-local is not the fastest overall (Euclidean is), it does produce the best quality. ANC-local is the only method that is able to adapt locally to varying energy landscapes and thus yields higher quality roadmaps. ANC-global is the second best in terms of quality but third in terms of time. ANC-local outperforms ANC-global.

**Fig. 7:**
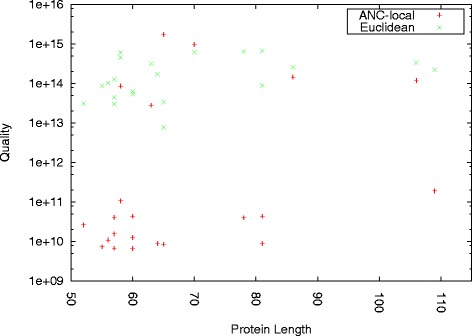
Quality as a function of protein length. ANC-local outperforms and its fastest competitor, Euclidean, in terms of quality irrespective of protein length

Figure 7 compares the quality of ANC-local to the quality of the fastest competitor, Euclidean. We see that regardless of protein length, ANC-local consistently outperforms Euclidean in terms of quality for most of the proteins studied. For the remaining proteins (distributed across the protein length range), the quality is similar. Recall that the aim is to generate pathways with low weight/energy. While computation time is important (and we have shown that ANC-local is competitive with other methods in this regard), it is more important to produce pathways of higher quality.

### Inspection of ANC-local learning choices

**Fig. 8:**
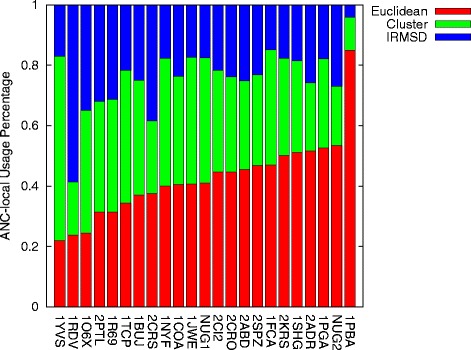
Connection method usage percentage in ANC-local across all proteins studied. Entries ordered by Euclidean usage

Figure 8 shows the percentage at which ANC-local used each individual connection method in constructing stable roadmaps for each protein. Entries are ordered by Euclidean usage as it is most often selected across the entire set.

For many proteins, ANC-local favors a single connection method, but for some (1O6X, 1TCP – NUG1), it favors 2 connection methods, and for 2 proteins (2PTL and 1R69), it selects equally among all connection methods. When it favors a subset of the connection methods, it selects the best individual method in both time and quality for 9 proteins, the best individual method in time only for 4 proteins, and the best individual method in quality only for 3 proteins.

## Conclusions

In this work, we present ANC-local, an algorithm that uses *local* learning to select appropriate connection methods in the context of PRM roadmap construction for protein folding. Our method monitors the performance and cost of various methods within the local neighborhood of the connecting conformation and adjusts their selection probabilities accordingly.

We have demonstrated a clear need for learning (i.e., ANC-global and ANC-local were the only methods to validate against all available experimental data) and showed that local learning is superior to global learning (i.e., ANC-local outperformed all other methods in terms of quality for 18 out of 23 proteins and was either the fastest or second fastest for 12 of the proteins). We also showed that our method produces a higher local planner success rate indicating that wise choices in how to use the costly local planner greatly impact performance. In many cases, ANC-local produces significantly higher quality results than the other methods. ANC-local removes the burden of deciding which method to use, leverages the strengths of the individual input methods, and it is extendable to include other future connection methods.
